# Epitope Mapping of Japanese Encephalitis Virus Neutralizing Antibodies by Native Mass Spectrometry and Hydrogen/Deuterium Exchange

**DOI:** 10.3390/biom14030374

**Published:** 2024-03-20

**Authors:** Jagat Adhikari, James Heffernan, Melissa Edeling, Estefania Fernandez, Prashant N. Jethva, Michael S. Diamond, Daved H. Fremont, Michael L. Gross

**Affiliations:** 1 Department of Chemistry, Washington University in St. Louis, Saint Louis, MO 63130, USA; jagat.adhikari@covanttx.com (J.A.); pjethva@wustl.edu (P.N.J.); 2 Department of Pathology and Immunology, Washington University School of Medicine, Saint Louis, MO 63130, USA; jheffernan@wustl.edu (J.H.); melissa.barrow@unimelb.edu.au (M.E.); e1fernandez@ucsd.edu (E.F.); mdiamond@wustl.edu (M.S.D.); fremont@wustl.edu (D.H.F.); 3 Department of Medicine, Washington University School of Medicine, Saint Louis, MO 63130, USA; 4 Andrew M. and Jane M. Bursky Center for Human Immunology and Immunotherapy Programs, Washington University School of Medicine, Saint Louis, MO 63130, USA; 5 Department of Molecular Microbiology, Washington University School of Medicine, Saint Louis, MO 63130, USA; 6 Department of Biochemistry and Molecular Biophysics, Washington University School of Medicine, Saint Louis, MO 63130, USA

**Keywords:** native MS, hydrogen/deuterium exchange, antibody/antigen interactions, monoclonal antibodies, epitope mapping

## Abstract

Japanese encephalitis virus (JEV) remains a global public health concern due to its epidemiological distribution and the existence of multiple strains. Neutralizing antibodies against this infection have shown efficacy in in vivo studies. Thus, elucidation of the epitopes of neutralizing antibodies can aid in the design and development of effective vaccines against different strains of JEV. Here, we describe a combination of native mass spectrometry (native-MS) and hydrogen/deuterium exchange mass spectrometry (HDX-MS) to complete screening of eight mouse monoclonal antibodies (MAbs) against JEV E-DIII to identify epitope regions. Native-MS was used as a first pass to identify the antibodies that formed a complex with the target antigen, and it revealed that seven of the eight monoclonal antibodies underwent binding. Native mass spectra of a MAb (JEV-27) known to be non-binding showed broad native-MS peaks and poor signal, suggesting the protein is a mixture or that there are impurities in the sample. We followed native-MS with HDX-MS to locate the binding sites for several of the complex-forming antibodies. This combination of two mass spectrometry-based approaches should be generally applicable and particularly suitable for screening of antigen–antibody and other protein–protein interactions when other traditional approaches give unclear results or are difficult, unavailable, or need to be validated.

## 1. Introduction

Japanese encephalitis virus (JEV) is a mosquito-transmitted flavivirus that affects approximately 68,000 persons annually worldwide [1]. JEV infections occur throughout the temperate and tropical regions of Asia, the western Pacific countries, and in northern Australia, locations where nearly half the world’s population resides [1,2]. The wide geographic distribution and the existence of multiple strains of the virus, coupled with the high mortality rate and neurological complications in survivors, makes JEV infection a serious public health problem. JEV is categorized by five genotypes (GI-V) that have distinct geographical and epidemiological distributions. Multiple vaccines exist for GIII, as it was formerly the predominantly circulating genotype, but there has been a shift in recent years towards circulation of GI, particularly in temperate and tropical climates [3].

Neutralizing antibodies have shown efficacy against JEV infection in vivo, and their titers often serve as correlates of protection [4,5,6]. Currently, more than 130 monoclonal antibodies (MAbs) and their derivatives are approved as therapeutics by the US Food and Drug Administration and the European Medicines Agency for the treatment of multiple pathologies, including infectious diseases [7,8]. Epitope identification is important for not only developing MAbs but also securing intellectual property. Those antigens that exhibit multiple binding sites provide an analytical challenge for determining the epitope. Structurally, the epitope is classified either as linear (a continuous stretch of amino acids that come into contact with the antibody) or conformational (two distinct regions of the antigen that make contact due to antigen folding with the MAb) [9]. Although X-ray crystallography [10], Nuclear Magnetic Resonance (NMR) [11], and CRYOgenic Electron Microscopy (Cryo-EM) [12] provide atomic-level resolution and remain the gold standards for epitope mapping [13], they are slow, expensive, and sometimes not applicable. Other methods include PEPSCAN (scanning of antigenic peptide library) [14], phage display library [15], alanine scanning mutagenesis [16,17], escape mutation analysis [18], and surface plasmon resonance (SPR) [19]. These latter methods have better throughput but are limited when the epitope is conformational or when structurally resolved measurements on the native antigen are needed.

Mass spectrometry-based techniques provide high sensitivity (requiring only nanogram and lower amounts of protein), good throughput (seamless connectivity between automated LC and ESI-MS instrumentation), medium structural resolution (peptide level and sometimes residue level), and compatibility with the native antigen. The application of MS began with epitope excision [20] and epitope extraction [21] as simple yet elegant MS approaches. Only the former can identify a conformational epitope. As the field progressed, hydrogen/deuterium exchange (HDX-MS) strategies [22,23,24,25], hydroxy-radical footprinting [26,27,28,29], and chemical crosslinking [30,31,32] were demonstrated for characterizing MAb-antigen complexes and protein–ligand binding interactions and providing complementarity to other biophysical approaches such as SPR. 

Native-MS has also emerged as a valuable approach to the characterization of intact noncovalent protein complexes [33,34]. Native-MS can provide binding stoichiometries and can rationalize different epitope regions for antibody–antigen binding experiments [35,36,37,38]. Stoichiometry of immune complexes affects both the neutralization mechanism and the pharmacokinetic behavior [39,40]. Additionally, native-MS can also provide important critical quality attribute information related to oligomerization, gas-phase stability (relatable to solution stability), truncation, and posttranslational modifications [41]. Recently, high-resolution native-MS was demonstrated to identify individual antibodies in a crude mixture of them [42,43]. A new approach termed direct-MS was recently proposed, in which direct native-MS analysis of antigen–antibody complexes can be achieved with little purification of serum samples [43]. In 2017, a native-MS based method called Intact Transition Epitope Mapping (ITEM) was introduced [44]. In ITEM, an immune complex of antigen fragments and an antibody is directly electrosprayed under native conditions, and binding assessed based on the dissociation extents of antigen fragments in the gas phase. A major drawback of this approach, however, is that only linear epitopes, not conformational epitopes, can be measured [45].

Experimentally, data acquisition and analysis of native-MS require only a few hours, although buffer exchange can slow the protocol. Native-MS can provide multidimensional data and serve as a “first line of offense” to determine the quality of expressed antigens and antibodies and to suggest their binding stoichiometry. Native-MS as a complementary approach is attractive for screening binding and for increasing the confidence of epitope mapping by SPR and mutagenesis because observing the molecular ions of the antibody, antigen, and a complex is reassuring. Our aim, however, is not to replace other traditional methods of binding but to show that rapid screening of MAbs binding to a target antigen by Native-MS can save experimental and instrument time before researchers embark on more demanding MS-based footprinting and/or HDX or other approaches to obtain an epitope map. Moreover, when coupled with ion mobility, native-MS can differentiate binding for different epitopes [46]. Some emerging MS methods that may impact epitope mapping are charge detection MS and mass photometry [47] and HDX approaches for both monoclonal and polyclonal antibodies [45,48].

Here, we describe a two-step approach that first utilizes native mass spectrometry (native-MS) to characterize the formation of a binding complex between several mouse monoclonal antibodies (MAbs) and an antigen, domain III of the envelope (E) protein of Japanese Encephalitis Virus (JEV E-DIII). E-DIII is an 11 kDa beta-sandwich domain consisting of seven beta strands that closely resemble a typical immunoglobulin-like fold. We then follow native-MS with HDX-MS analysis to locate the binding regions for several mouse anti-JEV MAbs. We present the native-MS data for five broadly neutralizing mouse MAbs (JEV-31, JEV-106, JEV-128, JEV-131, and JEV-143) and compare the outcome with previously reported HDX-MS and site-directed mutagenesis mapping data for key epitope regions [22]. We then describe native-MS and HDX-MS experiments for three additional MAbs (JEV-27, JEV-13, and JEV-142) and identify epitope regions distinct and distant from those of the five MAbs previously reported. Although biochemical and immunological data for JEV-27 were previously described [22], no biochemical, HDX-MS, or native-MS have been reported for JEV-13 and JEV-142. We take advantage of the complementary nature of these approaches: native-MS as a rapid screening tool and HDX-MS for the regional localization of epitopes in antibody-based therapeutics design, here illustrated for anti-JEV vaccine development.

## 2. Materials and Methods

### 2.1. Materials

Ammonium acetate, water, acetonitrile, formic acid, trifluoroacetic acid, guanidine hydrochloride, (tris(2-carboxy-ethyl)phosphine hydrochloride (TCEP.HCl) sodium hydroxide, phosphate-buffered saline (PBS), and Vivaspin 500 centrifugal molecular weight cut off filters were purchased from Sigma Aldrich (St. Louis, MO, USA). Deuterium oxide (D_2_O) was purchased from Cambridge Isotope Laboratories (Tewksbury, MA, USA). All the materials and reagents used were ACS grade and higher.

### 2.2. MAbs and JEV E-DIII

Generation of anti-JEV MAbs (JEV-27, JEV-31, JEV-106, JEV-128, JEV-131, and JEV-143) and JEV E-DIII expression and purification occurred as described previously [22]. An isotype control MAb, CHK-166, binds to the E1 protein of the different chikungunya virus and was reported previously [49]. JEV-13 and JEV-142 were generated from *Irf3^−/−^* mice after infection and boosting with JEV-SA14-14-2, as described previously [22]. Antibodies from hybridomas that bind to JEV-infected Vero cells were identified, separated by flow cytometry, and cloned by limiting dilution. IgGs from hybridoma supernatants were purified commercially (Bio X Cell, Lebanon, NH, USA) after adaptation for growth under serum-free conditions. The binding affinity of each of the MAbs generated against JEV E-DIII was measured by biolayer interferometry (BLI) using an Octet-Red96 device (Pall ForteBio, Menlo Park, CA, USA) in the manner described in [50].

### 2.3. Native Mass Spectrometry Analysis

To prepare samples for native-MS, the JEV E-DIII and MAb samples were buffer exchanged against 200 mM ammonium acetate solution in water. The desalting and buffer exchange involved five cycles of addition of 500 µL of 200 mM ammonium acetate solution to the samples and centrifugation at 15,000× *g* for 10 min at 4 °C by using Vivaspin 500 5 kDa MWCO filters (Sartorius, UK). The protein concentrations were determined by UV absorbance using a NanoDrop One spectrophotometer (Thermo Scientific, Wilmington, DE, USA). The JEV E-DIII (14 µM) and MAbs (7 µM) alone or 1:2 equivalents of MAb: JEV E-DIII ratio were incubated at room temperature for 1 h or more in 127 mM ammonium acetate (12.3 µL protein stocks in 200 mM ammonium acetate diluted with 7.3 µL of water (20 µL total)) before analysis with native-MS. 

For native-MS analysis, the JEV E-DIII and/MAb samples were directly infused into the ESI source of a Thermo Exactive Plus EMR Orbitrap mass spectrometer (Thermo Scientific, Waltham, MA, USA) operating in the positive-ion electrospray mode and utilizing commercial borosilicate emitters (ES380, Thermo Scientific, Hudson, NH, USA). The MS parameters were optimized to be a source voltage between 1.2–1.8 kV, capillary temperature of 55 °C, source DC offset of 25 V, injection flatapole of 8 ± 1 V, inter flatapole lens of 7 ± 1 V, bent flatapole DC of 6 ± 1 V, S-lens RF Level of 200, and CID and CE voltages at 100 and 200 V, respectively. The native-MS data were output from Xcalibur (Themo Scientific, Waltham, MA, USA) and the molecular weight was calculated using Microsoft Excel Version 2402 (Microsoft, Redmond, WA, USA) and plotted with Origin (OriginLab Corp., Northampton, MA, USA). The mass spectra were deconvoluted using the Protein Metrics (PMI, Cupertino, CA, USA) intact mass analysis module [51]. 

### 2.4. JEV E-DIII Intact Mass Measurement Using Regular Electrospray Ionization (ESI)

Intact mass measurements under denaturing ESI conditions were conducted with a 1:1 ratio of water and acetonitrile containing 0.1% formic acid and analyzed using a Maxis 4G QTOF instrument (Bruker, Billerica, MA, USA). The spectra were deconvolved by using MagTran [52]. 

### 2.5. Hydrogen/Deuterium Exchange Mass Spectrometry

HDX-MS experiments were performed as previously described [22]. Briefly, the MAbs were mixed in a 1:1 ratio with E-DIII, and HDX kinetic measurements were performed for 10, 30, 60, 120, 900, 3600, and 14,400 s. The HDX reaction was quenched with a solution containing 4 M guanidinium hydrochloride and 500 mM TCEP•HCL solution to the reaction vial at a 1:1 vol/vol ratio. The samples were injected for online protein digestion and conditions were optimized such that deuterium incorporation was measured consistently for 21 unique peptides spanning the 11-kDa JEV E-DIII protein. Apart from kinetic plot analysis, HDX data were analyzed statistically and plotted as Wood’s plots. The cumulative difference across all timepoint for each peptide was calculated. Also calculated was the propagated error for the cumulative differences. Only those peptides with cumulative difference of 99% confidence interval were highlighted in violet. Peptides with no significant differences were shown in gray.

## 3. Results and Discussion

### 3.1. Native-MS Analysis of Five MAbs That Bind to Lateral Ridge of JEV E-DIII

**Figure 1:**
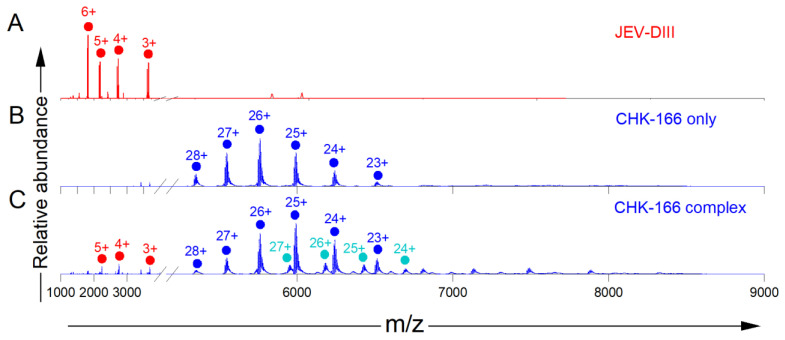
Native mass spectra of (**A**) JEV E-DIII, (**B**) CHK-166 MAb, and (**C**) CHK-166 MAb incubated with JEV E-DIII. Ions representing JEV E-DIII (red), CHK-166 alone (blue), and CHK-166 1:1 complex (cyan) are shown. The charge-state distribution in A is unusual and suggests some unfolding that would emphasize higher charge states, whereas the antigen in (**C**) likely originates by decomposition of the complex that protects the antigen during the spray and activation.

We carried out native-MS to confirm the binding of MAbs to JEV E-DIII. We chose an antigen and five antibodies that were previously shown to bind by HDX, ELISA, and alanine-mutagenesis experiments [22] as references. The binding affinities for each of the five MAbs as measured by BLI were in the range of 10 nM (Supplementary Table S1). We collected native mass spectra of the JEV E-DIII alone and when incubated with a control antibody, CHK-166 MAb, that does not bind to it but has specificity against Chikungunya virus (Figure 1). The native mass spectrum of JEV E-DIII revealed a narrow charge state distribution with a 6+ charged ion as the most abundant (Figure 1A). Two distinct series of peaks with similar charge-state distributions support the heterogeneity of the JEV E-DIII sample. Deconvolution of the native mass spectra showed two populations of the JEV E-DIII protein: one population of molecular weight (MW) 10,966.6 Da and another of 10,835.6 Da. The abundance ratio was 1:0.8, as determined from peak heights. To confirm the native-MS results of JEV E-DIII alone, we turned to denaturing MS and found evidence for two populations with approximately the same abundance ratio, confirming the heterogeneity (Supplementary Figure S1). The mass differences for the two groups of JEV E-DIII closely match the mass of a Met residue (131.1 Da), indicating a loss of Met from the N-terminus to provide the lower MW proteoform, as commonly observed for proteins. The experimental MWs for JEV E-DIII (with and without Met) from the mass spectra are 3 Da less than the theoretical masses calculated from the sequence of JEV E DIII (Supplementary Figure S1C) (10,969.51 Da and 10,838.3 Da), consistent with the presence of one disulfide bond [53]. 

The native mass spectrum of the CHK-166 antibody showed a clean and narrow charge-state distribution with most of the signals represented as six charge states centered around 26+ (Figure 1B, Supplementary Figure S2). The native mass spectra for the CHK-166 in the presence of the JEV E-DIII are similar to those acquired for CHK-166 alone (Figure 1C, Table S2, Supplementary Figure S2), showing mostly signals for free CHK-166 MAb and a small signal (~10%) of CHK-166 bound (160,656.8 Da peak) to a Met-truncated JEV E-DIII (10,820.1 Da). The difference in the calculated masses of CHK-166 (149,820.4 Da) and the most abundant ion cluster from a sample containing both CHK-166 and JEV E-DIII (149,836.7 Da) is only 16.3 Da, indicating that CHK-166 was unbound. This is consistent with that expected for the negative control antibody, CHK-166, which is known to bind Chikungunya virus structural proteins and E1 [54] and expected not to bind JEV E-DIII.

**Figure 2:**
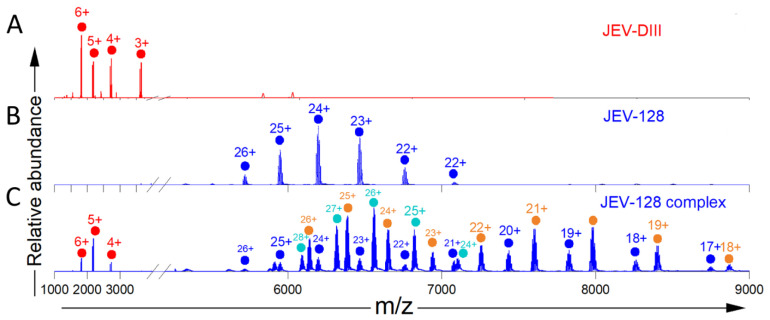
Native mass spectra for (**A**) JEV E-DIII as a reference (repeated from Figure 1), (**B**) JEV-128, and (**C**) JEV-128 incubated with JEV E-DIII. Ions representing different antibodies and antibody–antigen complexes are color coded blue (JEV-128 alone), orange (1:1 complex), and cyan (1:2 complex). The charge state distribution for JEV-DIII is unusual and suggests some unfolding.

Next, we acquired native-mass spectra of a MAb JEV-128 alone and in the presence of the JEV E-DIII (Figure 2, Supplementary Table S2, Figure S2). JEV-128, as shown previously using both biochemical assays and HDX, binds at the lateral ridge region of JEV E-DIII [22]. The mass spectrum of the JEV-128 MAb showed a series of peaks representing different charge states centered around 24+ (Figure 2B). Deconvolution of the mass spectra indicates that JEV-128 MAb has a MW of 148,753.9, which matches well that of a typical MAb. Upon incubation with the JEV E-DIII antigen, the abundance of the free MAb becomes smaller, and additional charge state distributions at higher *m/z* (Figure 2C) appear. Although the charge state distribution for JEV-128 is homogeneous (blue dots in Figure 2B), three charge state distributions appear when it is mixed with JEV E-DII. The charge state distribution for two ions (blue dots and orange dots) are bimodal, whereas the third is unimodal (green dots). The bimodal distribution may be due to increased flexibility induced at the unoccupied site followed by recovered stabilization upon the second binding of the antigen. These distributions are also seen in spectra presented later. Deconvolution yielded three experimental masses: 148,755.8, 159,577.2, and 170,555.0 Da. The ions with a MW of 148,755.8 Da have the mass of a free JEV-128 MAb, and the measured MW is in good agreement with the MW from the native mass spectrum of the JEV-128 alone. The higher mass species is consistent with 1:1 (159,577.4 Da) and 1:2 (170,555 Da) JEV E128: JEV E-DIII complexes, respectively. The 1:2 complex contains both full-length and truncated JEV E-DIII bound with the JEV-128 MAb, whereas the 1:1 complex only shows the truncated bound species, possibly because it has already transitioned to 1:2. We found no signal corresponding to the dimer in the mass spectra of the JEV E-DIII alone. Furthermore, the absence of higher-order binding stoichiometries for JEV E-DIII binding to JEV-128 strongly suggests that JEV E-DIII remains monomeric under native-MS conditions, showing a binding stoichiometry of one JEV E-DIII monomer per Fab region of MAb or a 1:2 complex, as expected based on the starting molar stoichiometric ratio at incubation.

Although a 1:2 complex based on the starting concentrations of the incubated JEV E DIII and the MAbs was introduced to the gas phase of the instrument, native-MS also showed a 1:1 bound complex, the free MAb, and the antigen. We cannot be certain that these ions represent antibody, antigen, and 1:1 complexes in solution, as they may originate from in-source collisional activation. In-source collisional activation easily dissociates noncovalent complexes held together by hydrophobic interactions [37,55]. Our attempts to reduce the dissociation of the complex by lowering the collision energy and to obtain meaningful information were not successful, instead providing mass spectra characterized by poor signal-to-noise ratios.

**Figure 3:**
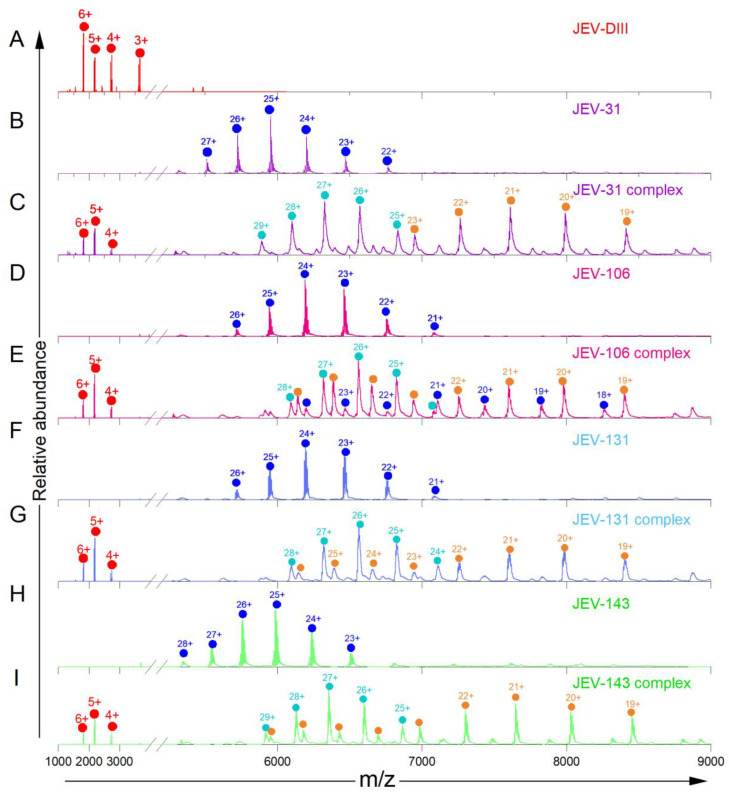
Native mass spectra of MAb alone and in the presence of JEV E-DIII for JEV-31 (**B**,**C**), JEV-106 (**D**,**E**), JEV-131 (**F**,**G**), and JEV-143 (**H**,**I**), respectively. The native mass spectrum of JEV E-DIII, shown as references (repeated from Figure 1) (**A**). Different species are color coded red (JEV E-DIII alone), blue (antibody alone), orange (1:1 complex), and cyan (1:2 complex). See Figure 2 for the mass spectrum of JEV-DIII.

We then carried out native-MS experiments with four additional MAbs (JEV-31, JEV-106, JEV-131, and JEV-143), which were previously reported to be lateral-ridge-binding MAbs with strong protection for regions in the N-terminal region and in the A strand (residues 304 to 310), BC loop (residues 326 to 342), and DE loop (residues 355 to 371) of E-DIII, regions that correspond to the well-defined lateral ridge (LR) in HDX-MS data [22]. Comparisons of the native mass spectral pairs (i.e., in the absence or the presence of the JEV E-DIII) show clear charge distributions for the MAbs alone and new charge distributions at higher *m/z* for the antibody–antigen (JEV E-DIII) complexes (Figure 3, Supplementary Table S2, and Figure S2). Like the native mass spectrum of the JEV-128 complex, those of the other MAbs also demonstrate the existence of two stoichiometric ratios of bound complexes (1:1 and 1:2), as determined from experimental MWs. It is important to note that the preparation and acquisition of each sample took approximately 1 h, and speed can be further enhanced by parallel processing. Native-MS rapidly and clearly confirms complex formation of the antigen and antibody.

### 3.2. JEV-27 mAb Does Not Bind JEV E-DIII

**Figure 4:**
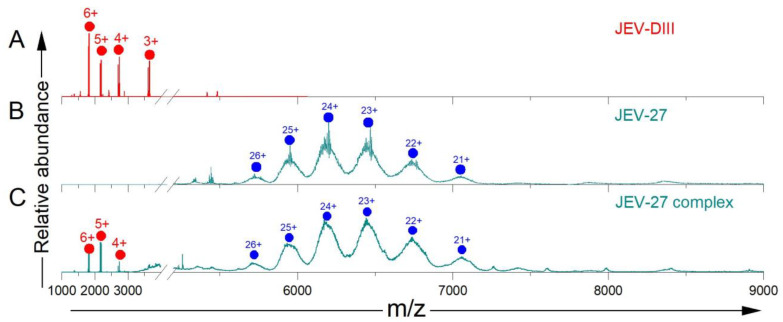
The JEV E-DIII spectrum is shown on the top panel as a reference (repeated from Figure 1) (**A**). Native-MS analysis of JEV-27 alone (**B**) and in the presence of the JEV E-DIII antigen (**C**). Different species are color coded red (JEV E-DIII alone) and blue (antibody alone). The small signals and the baseline “hump” around *m/z* 4000 were not identified, but they are likely due to an impurity.

We then asked whether another MAb, JEV-27, binds with JEV E-DIII, which showed weak neutralizing activity with antibody titers for various strains [22]. We directly infused JEV-27 alone and with JEV E-DIII and found no significant difference with and without JEV E-DIII, indicating the absence of a complex (Figure 4, Supplementary Table S2, and Figure S2). The MWs calculated from the charge state series for JEV-27 alone and for JEV-27 incubated with JEV E-DIII are 148,766.2 Da and 148,463 Da, respectively. The difference in the calculated MWs of the major species is 303.2 Da, much less than the MW of the JEV E-DIII alone, indicating that no binding of the antigen occurs.

The peaks for JEV-27 are broad, likely because they are composite, convolved with interfering peaks from other antibodies of slightly different MWs or of antibodies containing heterogeneous glycosylation. This makes the accurate calculation of the MWs of the JEV-27 from the native mass spectra acquired here susceptible to errors. The spectra also revealed truncated MAbs or other contaminating proteins at lower *m/z* (extra peaks around *m/z* 5000). The outcome is consistent with no binding observed with BLI measurements of binding affinity (Supplementary Table S1). Nevertheless, the understanding of binding behavior and sample quality not only saves time when performing complicated experiments but also provides guidance for future protein expression and purification. These conclusions would be difficult to draw on the basis of Western blot or ELISA measurements.

**Figure 5:**
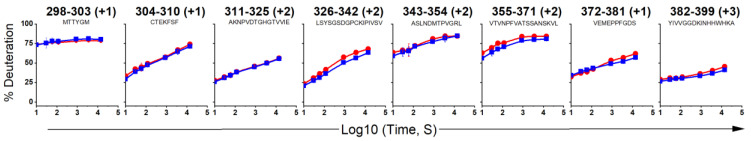
HDX-MS analysis of JEV-27 alone (red circles) and in the presence of the JEV E-DIII antigen (blue squares). For HDX-MS kinetics, measurements for each time point were performed in duplicate, and the data are representative of two independent experiments. The standard deviations not seen are smaller than the data points. The differences are not significantly different, as shown by the statistical analysis associated with Wood’s plots in SI, Figure S2.

One course of action upon obtaining native mass spectra would be to cancel the more detailed and time-consuming HDX experiments. To ensure that we are not misled by the non-binding behavior at the protein level, as seen by native-MS, we continued with an HDX experiment coupled with pepsin digestion for JEV-27 with JEV E-DIII. A general summary of the HDX-MS data and experimental conditions is provided (Supplementary Table S3). All the peptides covering the entire JEV E-DIII showed little or no difference in the average deuterium uptake with or without the antibody (Figure 5 and Supplementary Table S4). Thus, HDX and native-MS data together confirm the non-binding behavior of JEV-27 MAb. Lack of binding is consistent with the weak neutralization activity observed for JEV-27 against different strains of JEV [22]. The native mass spectra also reflect the quality of the sample, serving to screen samples rapidly for further study by HDX or other footprinting.

### 3.3. Native-MS and HDX-MS for Epitope Mapping

**Figure 6:**
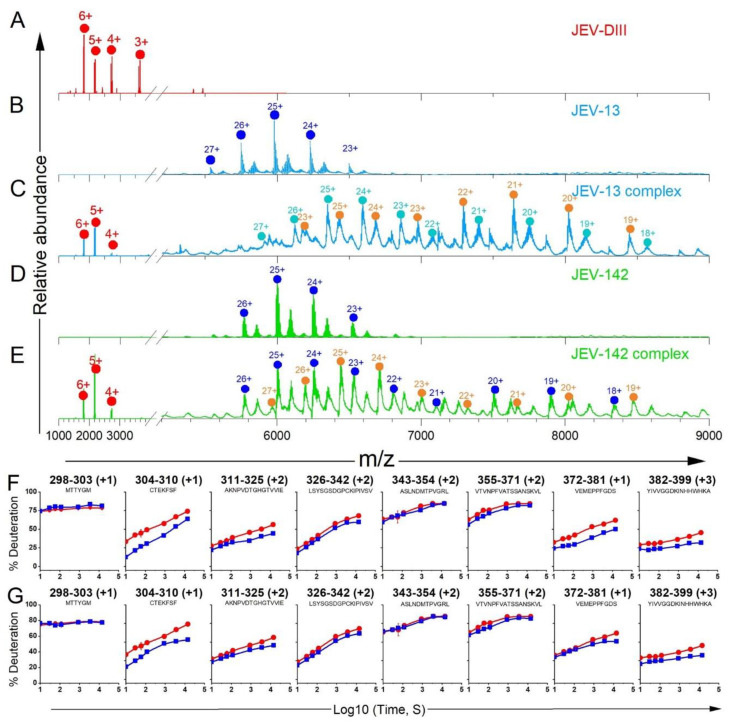
Native mass spectra of (**A**) JEV E-DIII only (repeated from Figure 1), (**B**) JEV-13 MAb only, (**C**) JEV-13 incubated with JEV E-DIII, (**D**) JEV-142 MAb only, and (**E**) JEV-142 incubated with JEV E-DIII, respectively. Different species are color coded red (JEV E-DIII alone) blue (antibody alone), orange (1:1 complex), and cyan (1:2 complex). The HDX kinetic plots for various peptide regions of the antigen upon binding with (**F**) JEV-13 and (**G**) JEV-142, respectively: unbound JEV E-DIII (red circles) and bound with MAbs (blue squares). For HDX-MS, kinetic measurements for each time point were performed in duplicate, and the data are representative of two independent experiments. Those deviations not seen graphically are smaller than the data points.

We explored the binding of two new anti-JEV MAbs JEV-13 and JEV-142 by using native-MS to screen followed by HDX-MS to locate the epitope regions. The native mass spectra of the MAb alone for JEV-13 and JEV-142 resulted in peaks centered around *m/z* 6000 (Figure 6B,D) and another series of peaks at lower intensity that afforded slightly higher MWs for both samples, suggesting the presence of interfering antibody(ies) in the sample. Moreover, the native mass spectra of JEV E-DIII in mixture with both JEV-13 and JEV-142 showed charge states at higher *m/z’*s consistent with complex formation (Figure 6C,E, Supplementary Table S2 and Figure S2). The MWs derived from these peaks correspond well with those expected for the formation of the complexes, indicating binding of each of the anti-JEV MAbs with JEV E-DIII.

**Figure 7:**
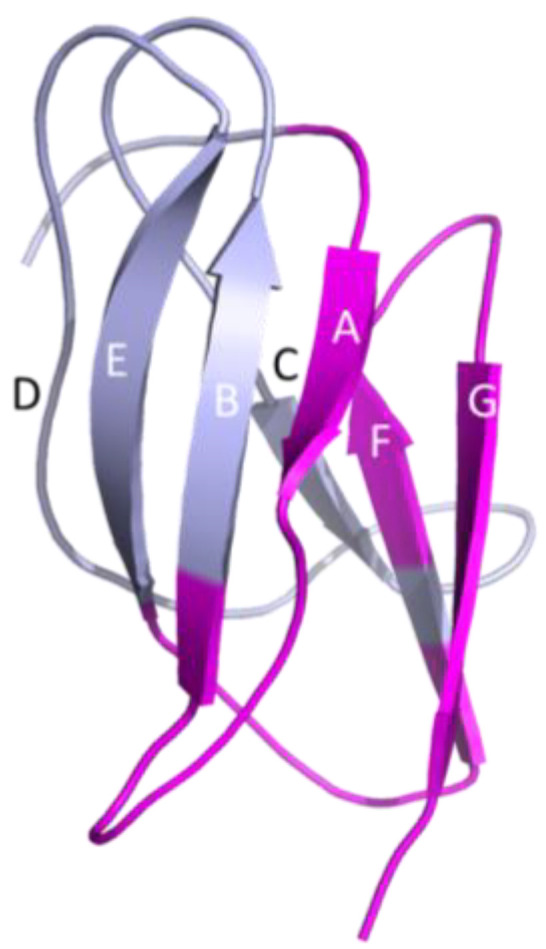
HDX-MS data for JEV-13 and JEV-142 mapped onto the crystal structure of DIII of JEV envelope protein (PDB: 3P54)^50^. The letters A–G on the crystal structure model represent seven beta strands of JEV E-DIII. The regions in the JEV E-DIII with increased protection are colored in magenta. The HDX-MS data and location of the epitope regions for other MAbs were previously described (Figure 4A,C [22]).

To obtain site-specific binding information for the new anti-JEV MAb complexes, we employed HDX and compared free JEV E-DIII with MAb: JEV E-DIII complexes for MAbs JEV-13 and JEV-142. HDX as a function of time for JEV E-DIII alone and with MAbs reveals those regions in JEV E-DIII with distinct differences upon binding (Figure 6F,G and Supplementary Table S4). For the binding of JEV-13, the deuterium uptake is notably reduced for regions in the A-strand for the peptide 304–310. Peptides covering the AB, EF, and FG loops, namely 311–325, 372–381, and 382–399, respectively, also show strong protection (Figure 6F and Figure 7, Supplementary Table S4), pointing to these locations as the binding sites for JEV-13. The confidence in the observed HDX behavior is increased because the differences in these regions are from replicate experiments, and the behavior observed with overlapping peptides is consistent.

For JEV-142 binding to JEV E-DIII, the most significant differential reduction in deuterium uptake occurs in similar regions as for JEV-13. The greatest deuterium uptake reduction is seen in the A-strand for the peptide 304–310 (Figure 6G and Figure 7), which is strongly protected in the presence of JEV-142. Peptides covering the AB, EF, and FG loops, namely 311–325, 372–381, and 382–399, respectively, also show a decrease in deuterium incorporation upon binding, although some of the differences are less pronounced than for JEV-13 (Figure 6G, Supplementary Table S4). There is little change for the BC (326–342) and DE (355–371) loop regions. JEV-13 and JEV-142 share a common binding region that mainly affects the A-strand and neighboring regions of the JEV E-DIII and is represented in magenta in JEV E-DIII portion from crystal structure of JEV envelope protein (PDB: 3P54 [56]) (Figure 7). This region marks a different and distant epitope located near the A-strand and away from the previously known lateral ridge epitope in the vicinity of the N-terminus, BC, and DE loop regions which are classified as A-strand binders.

## 4. Conclusions

We explored two MS-based approaches used to identify MAbs that bind with the antigen, JEV E-DIII, and subsequently the epitope regions of JEV E-DIII binding to several anti-JEV Mabs, not as replacements for conventional approaches but as complements. Native-MS revealed that all anti-JEV MAbs except JEV-27 bind up to two monomers of JEV E-DIII molecules. Because co-crystallization for JEV E-DIII with MAbs is difficult, we then used HDX to determine the epitopes of anti-JEV MAbs. JEV-13 and JEV-142 share the same epitope near the A-strand regions of JEV E-DIII.

From a methodological point of view, our data demonstrate that native-MS and HDX-MS done in series provide a valuable approach for epitope mapping of antigen–antibody interactions with the peptide-level resolution. The workflow can generally be applied to other systems for which a high-resolution structure of the antibody–antigen complex is not available or when higher throughput capabilities provided by native-MS are needed. Observations of ions with appropriate *m/z’*s reassures any workflow that employs other tools.

## Data Availability

Analyzed data for HDX-MS kinetics and native MS *m/z* peaks presented in this work are provided as tables and figures in the supplementary information. Mass spectrometry raw files can be provided upon request.

## Supplementary Materials

The following supporting information can be downloaded at https://www.mdpi.com/article/10.3390/biom14030374/s1, Figure S1: Mass spectrum of JEV E-DIII acquired under (A) native ESI, (B) denaturing electrospray; deconvoluted mass spectrum (left) and raw spectra (right). (C) Sequence of JEV E-DIII. JEV E-DIII contains one disulfide bond. The difference in mass, 10,966.6 Da (red circles), 10,835.6 Da (blue stars), is 131.1 Da the mass of an initiator methionine at the N terminus.; Figure S2: Deconvoluted Native-MS spectra for JEV-DIII in the absence and presence of different antibodies. Mass spectra are deconvoluted using Protein Metrics (PMI) intact mass analysis module; Figure S3: Differential Wood’s plot for the JEV DIII in the presence and absence of antibodies JEV-27, JEV-13, and JEV-142. Cumulative sum of differences across all HDX time points for each peptide was calculated. Based on propagated error in HDX measurements, statistically protected peptides were highlighted. The gray bars depict peptides where there is no significant change in the bound vs. unbound states (*p* = 0.01), while violet bars indicate peptides exhibiting protection upon binding to antibody; Table S1: Binding affinity measurements of each of the MAbs generated against JEV E-DIII was measured by biolayer interferometry using an Octet-Red96 device (Pall ForteBio); Table S2: List of the *m/z* values and the molecular weights obtained for the JEV-E-DIII and the antibodies from native-MS spectra; Table S3: HDX-MS data summary; Table S4: Peptic peptides for the JEV E DIII alone vs. with various MAbs. The deuterium uptake (%) for each of the experiments is listed.
