# Supplementary material for: Epitope Mapping of Japanese Encephalitis Virus Neutralizing Antibodies by Native Mass Spectrometry and Hydrogen/Deuterium Exchange
Source: Biomolecules. 2024 Mar 20;14(3):374. doi: 10.3390/biom14030374 (PMC10967844; doi:10.3390/biom14030374)
Supplement: Supplementary file 1 [file biomolecules-14-00374-s001.zip › 2024JEV-nativeMS-HDX-SI-Rev10.pdf]

Supporting Information

Epitope mapping of Japanese encephalitis virus neutralizing antibodies by native mass spectrometry and hydrogen deuterium exchange

Jagat Adhikari,[1,7] James Heffernan,[2] Melissa Edeling,[2] Estefania Fernandez,[2] Prashant N. Jethva[1], Michael S. Diamond,[2,3,4,5] Daved H. Fremont,[2,5,6] Michael L. Gross[1]

1Department of Chemistry, Washington University in St. Louis, Saint Louis, MO 63130, USA; jagat.adhikari@covanttx.com (J.A.); pjethva@wustl.edu (P.N.J.)

2Department of Pathology and Immunology, Washington University School of Medicine, Saint Louis, MO 63130, USA; jheffernan@wustl.edu (J.H.); melissa.barrow@unimelb.edu.au (M.E.); e1fernandez@ucsd.edu (E.F.); mdiamond@wustl.edu (M.S.D.); fremont@wustl.edu (D.H.F.)

3Department of Medicine, Washington University School of Medicine, Saint Louis, MO 63130, USA

4Andrew M. and Jane M. Bursky Center for Human Immunology and Immunotherapy Programs, Washington University School of Medicine, Saint Louis, MO 63130, USA

5Department of Molecular Microbiology, Washington University School of Medicine, Saint Louis, MO 63130, USA

6Department of Biochemistry and Molecular Biophysics, Washington University School of Medicine, Saint Louis, MO 63130, USA

*Correspondence: mgross@wustl.edu

†Current Address: Covant Therapeutics, Boston, MA 02210, USA.

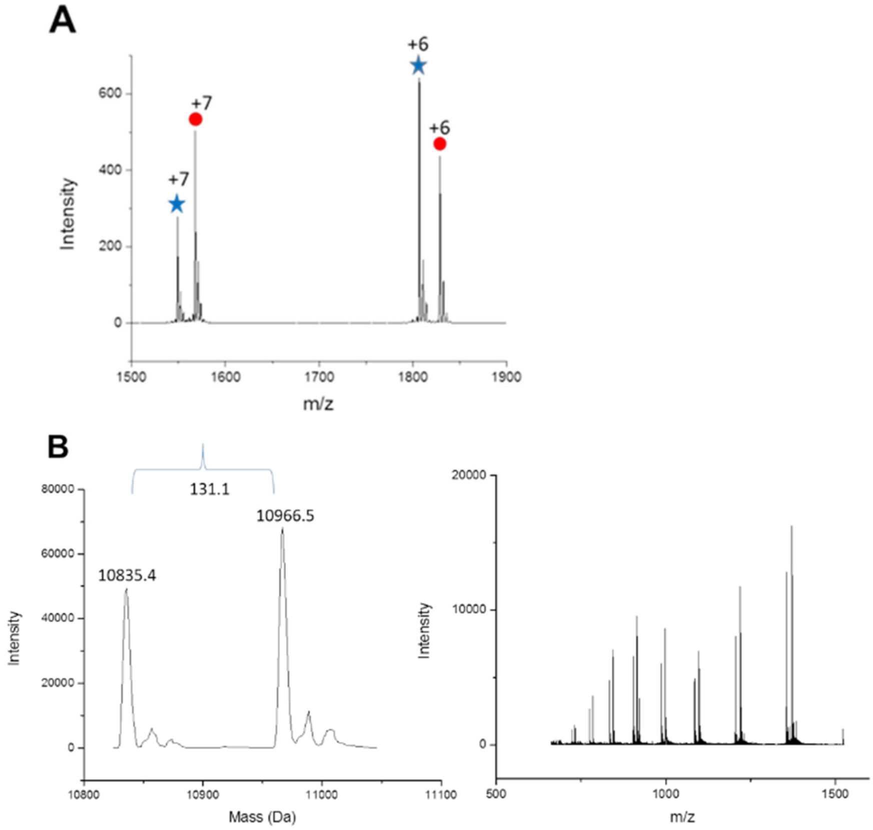

C Sequence of JEV E-DIII

MTTYGMCTEK FSFAKNPVDT GHGTVVIELS YSGSDGPCKI PIVSVASLND
MTPVGRLVTV NPFVATSSAN SKVLVEMEPP FGDSYIVVGR GDKQINHHWH KA

Figure S1. Mass spectrum of JEV E-DIII acquired under (A) native ESI, (B) denaturing electrospray; deconvoluted mass spectrum (left) and raw spectra (right). (C) Sequence of JEV E-DIII. JEV E-DIII contains one disulfide bond. The difference in mass, 10966.6 Da (red circles), 10835.6 Da (blue stars), is 131.1 Da the mass of an initiator methionine at the N terminus.

Table S1. Binding affinity measurements of each of the MAbs generated against JEV E-DIII was measured by biolayer interferometry using an Octet-Red96 device (Pall ForteBio).

	JEV MAb	k_a	k_d	K_d (nM) SS	K_d (nM) Kinetic	$t_{1/2}$ (s)
1	JEV E13	3.18e5	8.53e-4	7	2.7	1820
2	JEV E31	6.39e4	1.95e-3	63	31	383
3	JEV E106	4.63e4	1.02e-3	73	22	267
4	JEV E128	8.65e4	1.83e-3	43	21	355
5	JEV E131	1.1e5	1.35e-3	32	21	320
6	JEV E143	3.79e4	1.43e-3	23	38	330
7	JEV E27			No binding		

Table S2. List of the m/z values and the molecular weights obtained for the JEV-E-DIII and the antibodies from native-MS spectra.

Sample	Complex status	Charge state (z)												moleculer weight
		18	19	20	21	22	23	24	25	26	27	28	29	
JEV DIII	Unbound	1806.825 **(z6)**	2168.025 **(z5)**	2709.695 **(z4)**	3612.83 **(z3)**									10836.0 Da
		1828.730 **(z6)**	2194.115 (z5)	2742.640 (z4)	3656.480 (z3)									10967.0 Da
CHK-166	Unbound						6515.3	6243.82	5993.865	5763.125	5549.895	5351.63		149820.4 Da
	1:1 complex (Unbound)					6811.465	6515.415	6244.05	5993.94	5763.335	5549.81			149836.7 Da
	1:1 complex (~10%)							6695.305	6427.245	6180.235	5951.06			160656.5 Da
JEV-128	Unbound				7084.475	6762.17	6468.28	6198.695	5950.84	5721.665				148753.9 Da
	Unbound	8264.84	7829.75	7438.265	7083.995	6762.465	6468.97	6199.3	5951.565	5722.075				148755.8 Da
	1:1 complex	8875.345	8400.0000	7979.745	7599.67	7253.735	6939.105	6650.17	6383.75	6138.49	5911.225			159577.2 Da
	1:2 complex							7107.72	6823.24	6561.05	6317.5	6092.22	5881.775	170555.0 Da
JEV-31	Unbound					6764.5400	6469.7300	6200.0150	5951.9050	5723.0800	5511.0750			148778.5 Da
	1:1 complex		8412.8250	7992.2850	7611.9950	7265.7250	6949.2650	6660.185	6393.405	6148.585				159830.1 Da
	1:2 complex			8542.34	8136.9	7764.085	7429.72	7119.345	6833.9150	6569.8550	6327.7300	6101.0900	5890.2450	170808.0 Da
JEV-106	Unbound				7084.41	6754.79	6460.885	6191.755	5943.885	5715.505				148580.8 Da
	1:1 complex							7078.81	6825.09	6562.56	6318.93	6093.475		159602.7 Da
	1:2 complex						7417.94	7109.42	6825.09	6562.56	6318.93	6093.475		170589.0 Da
JEV 131	Unbound				7087.295	6757.425	6463.35	6194.07	5946.06	5723.47				148636.7 Da
	1:1 complex	8870.195	8402.94	7983.125	7602.815	7257.13	6950.085	6660.47	6395.29	6141.685	5914			159640.7 Da
	1:2 complex				8125.045	7757.295	7419.435	7110.645	6825.915	6563.57	6321.145	6094.07	5883.185	170620.0 Da
JEV-143	Unbound						6511.955	6240.54	5991.08	5760.425	5546.825	5348.685		149747.8 Da
	1:1 complex		8452.94	8030.13	7648.03	7300.225	6983.145	6698.915	6431.4	6177.99	5955.17			160587.5 Da
	1:2 complex						7460.49	7149.93	6864.025	6600.015	6355.75	6128.555	5917.04	171563.4 Da
JEV-27	Unbound				7071.47	6734.51	6469.27	6199.62	5951.715	5723.305				148766.2 Da
	No complex formed			7111.72	6773.79	6477.665	6207.5							~148000.0 Da

Figure S2: Deconvoluted Native MS spectra for JEV-DIII in the absence and presence of different antibodies. Mass spectra are deconvoluted using Protein Metrics (PMI) intact mass analysis module.

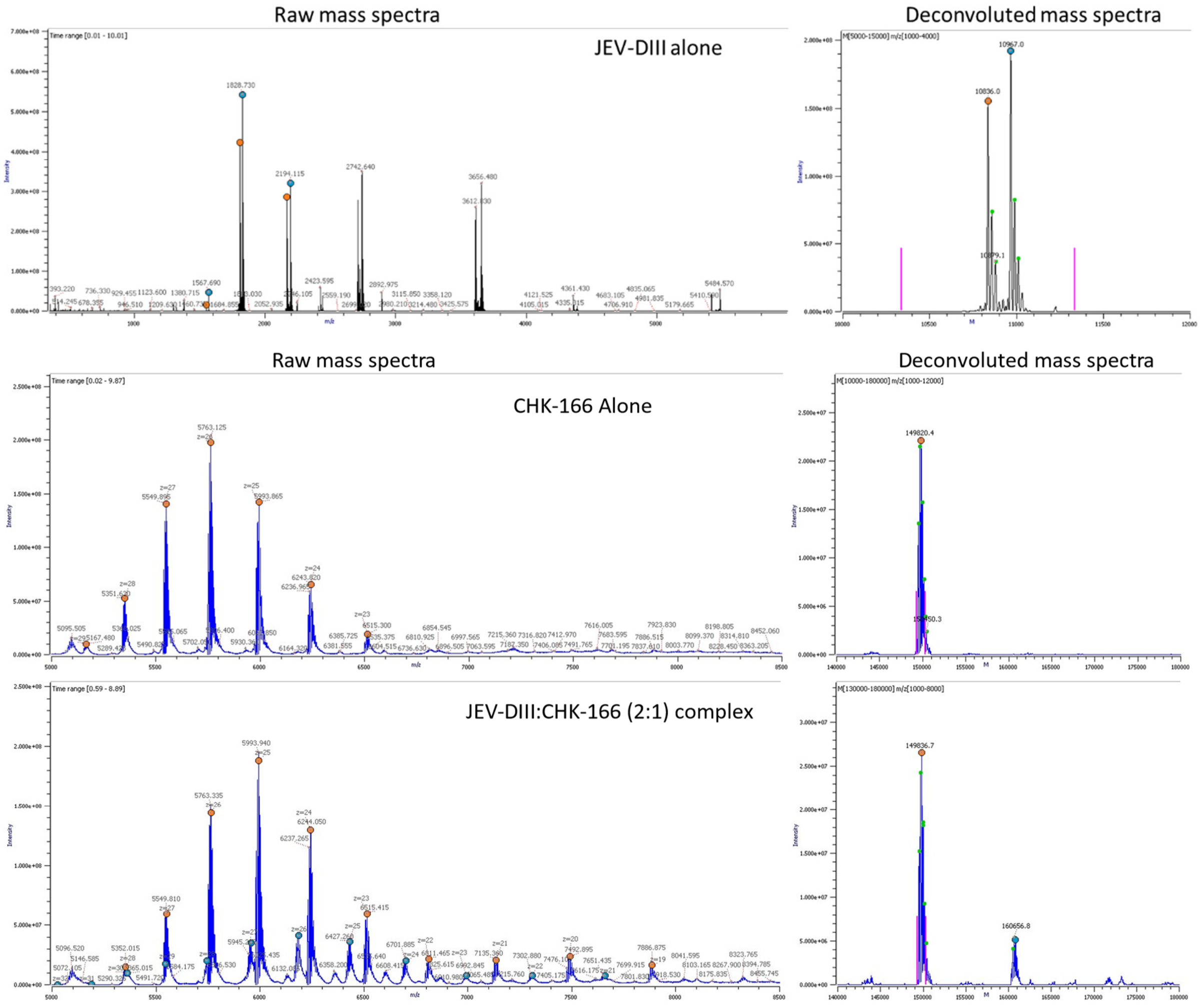

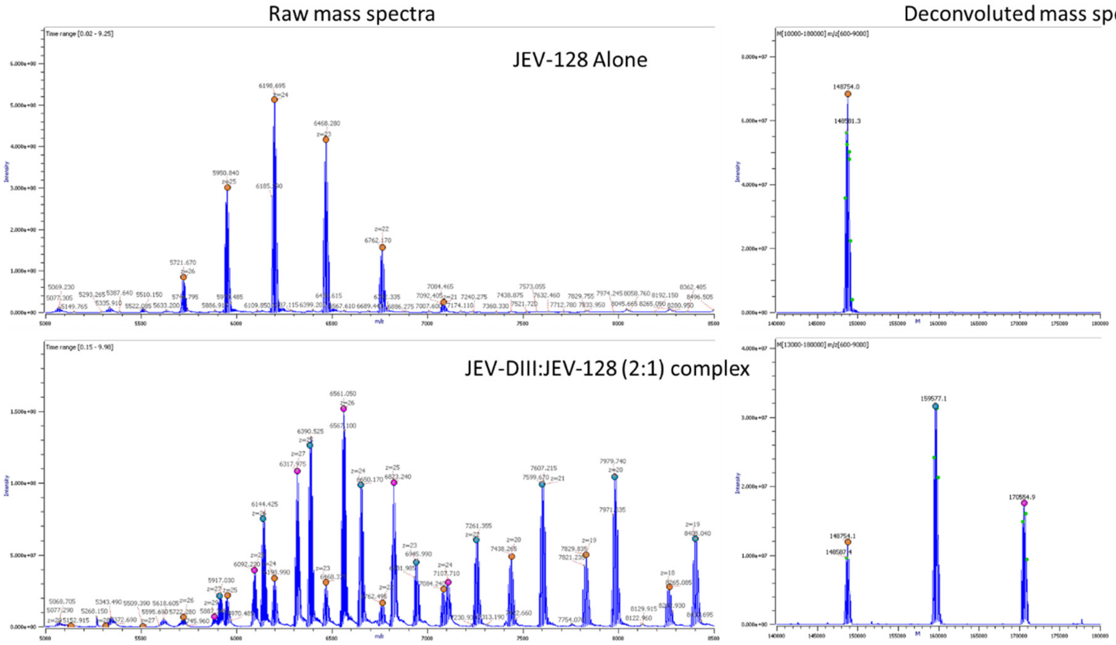
Raw mass spectra
Deconvoluted mass spectra
JEV-128 Alone
JEV-DIII:JEV-128 (2:1) complex
148754.0
159577.1
170554.9

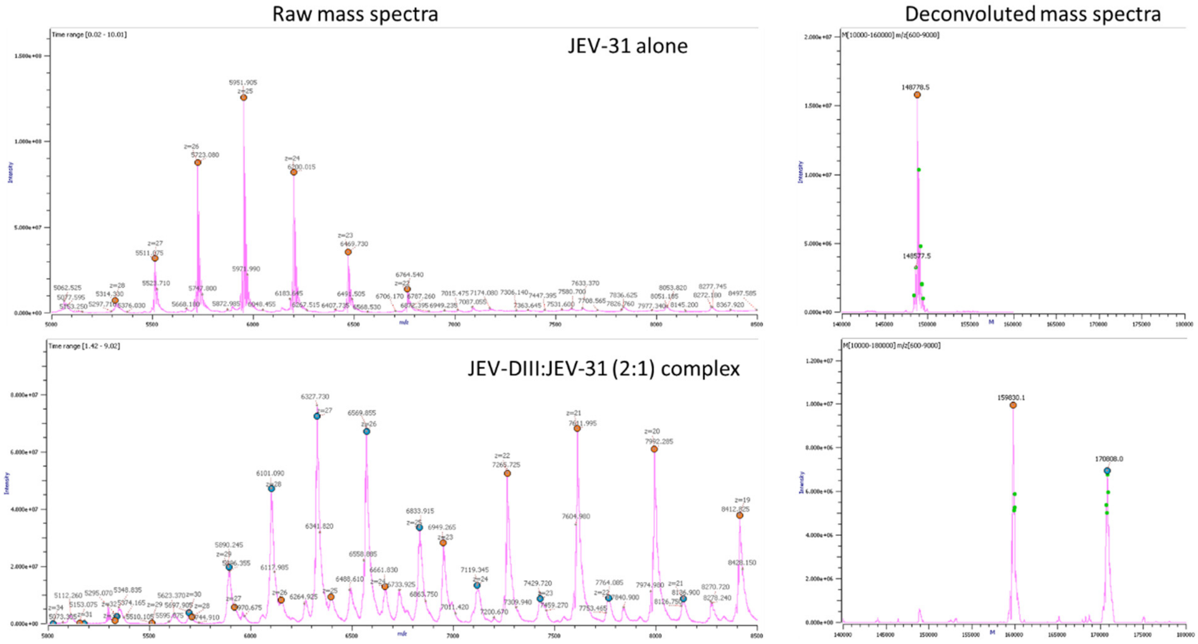
Raw mass spectra
Deconvoluted mass spectra
JEV-31 alone
JEV-DIII:JEV-31 (2:1) complex
148778.5
159830.1
170808.0

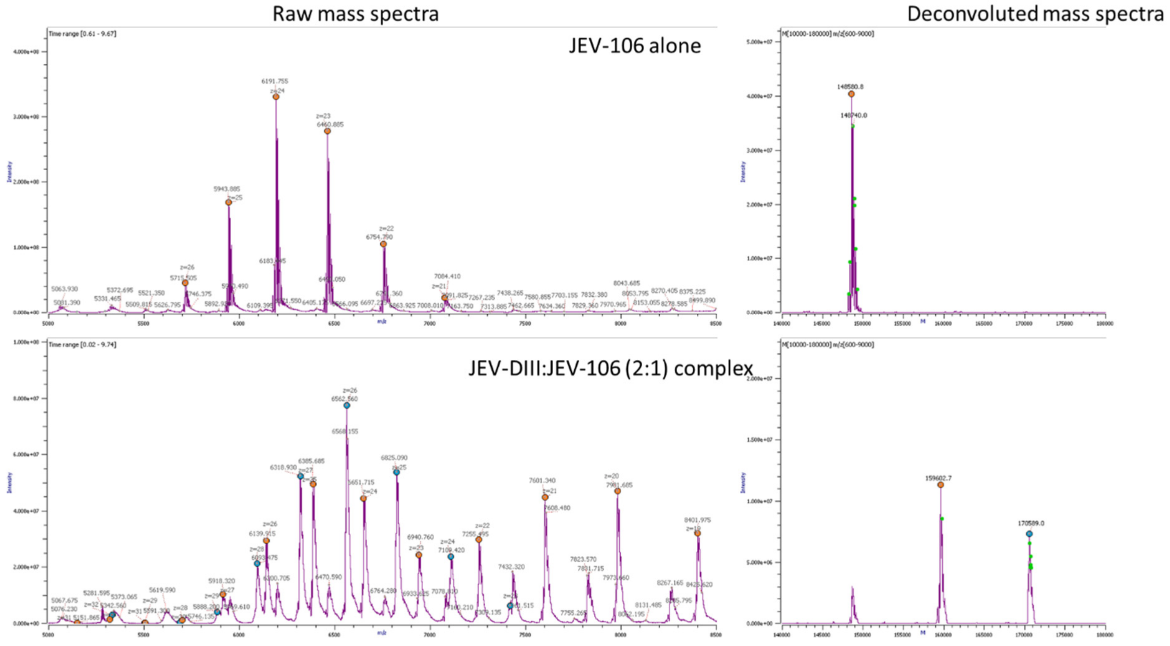
Raw mass spectra
Deconvoluted mass spectra
JEV-106 alone
JEV-DIII:JEV-106 (2:1) complex

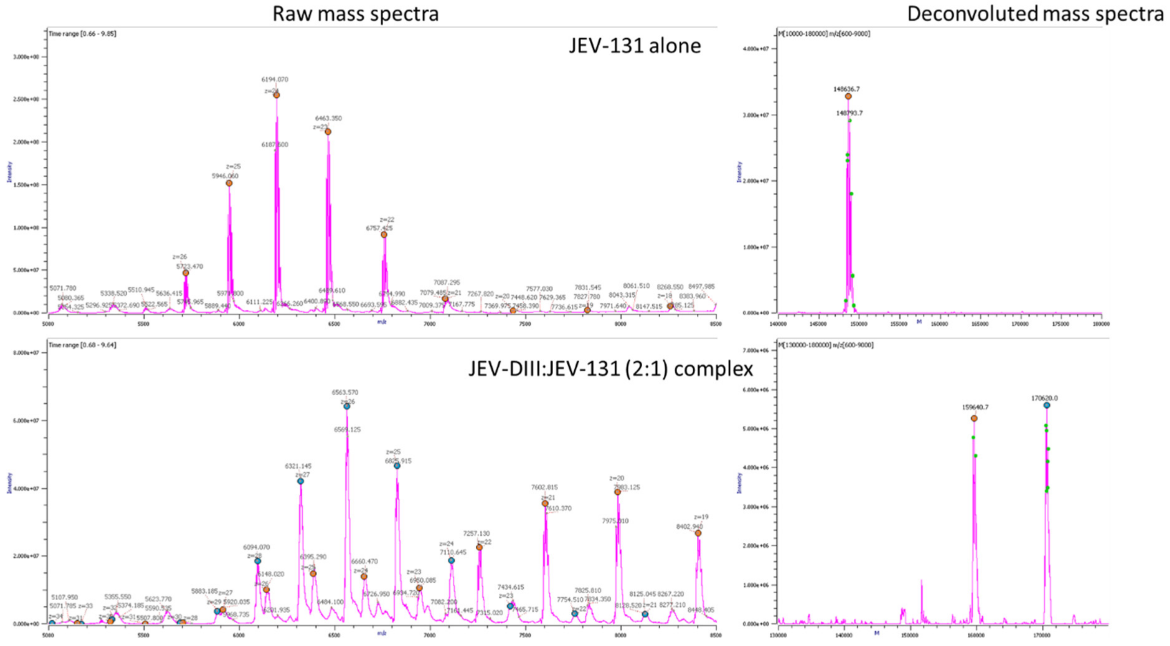
Raw mass spectra
Deconvoluted mass spectra
JEV-131 alone
JEV-DIII:JEV-131 (2:1) complex

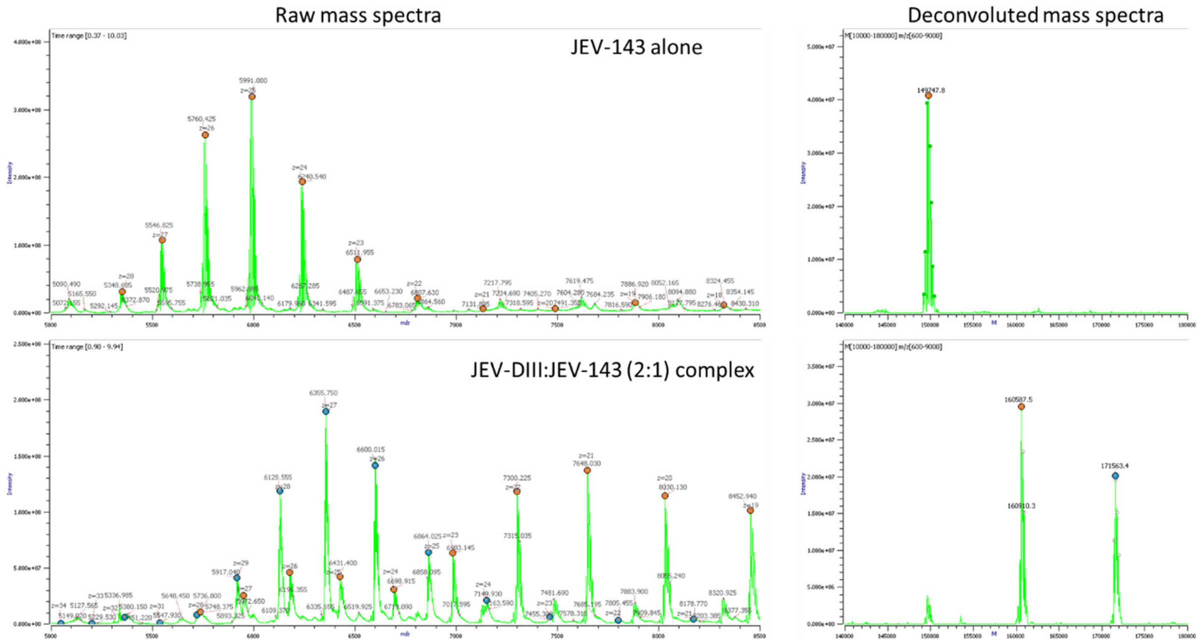
Raw mass spectra
Deconvoluted mass spectra
JEV-143 alone
JEV-DIII:JEV-143 (2:1) complex

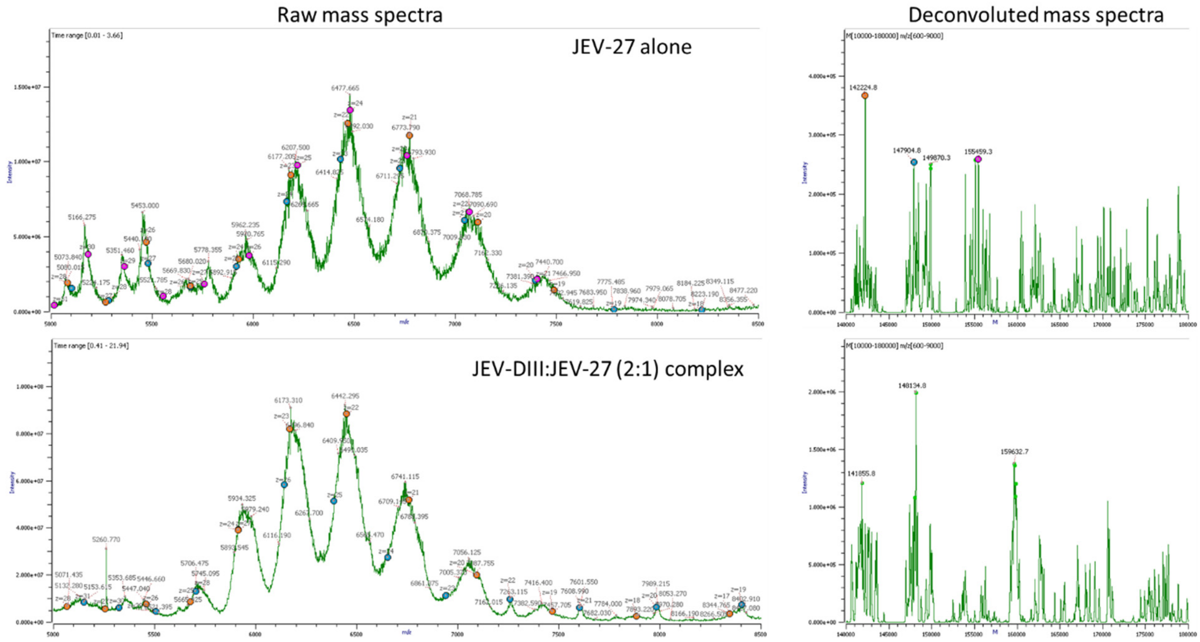
Raw mass spectra
Deconvoluted mass spectra
JEV-27 alone
JEV-DIII:JEV-27 (2:1) complex

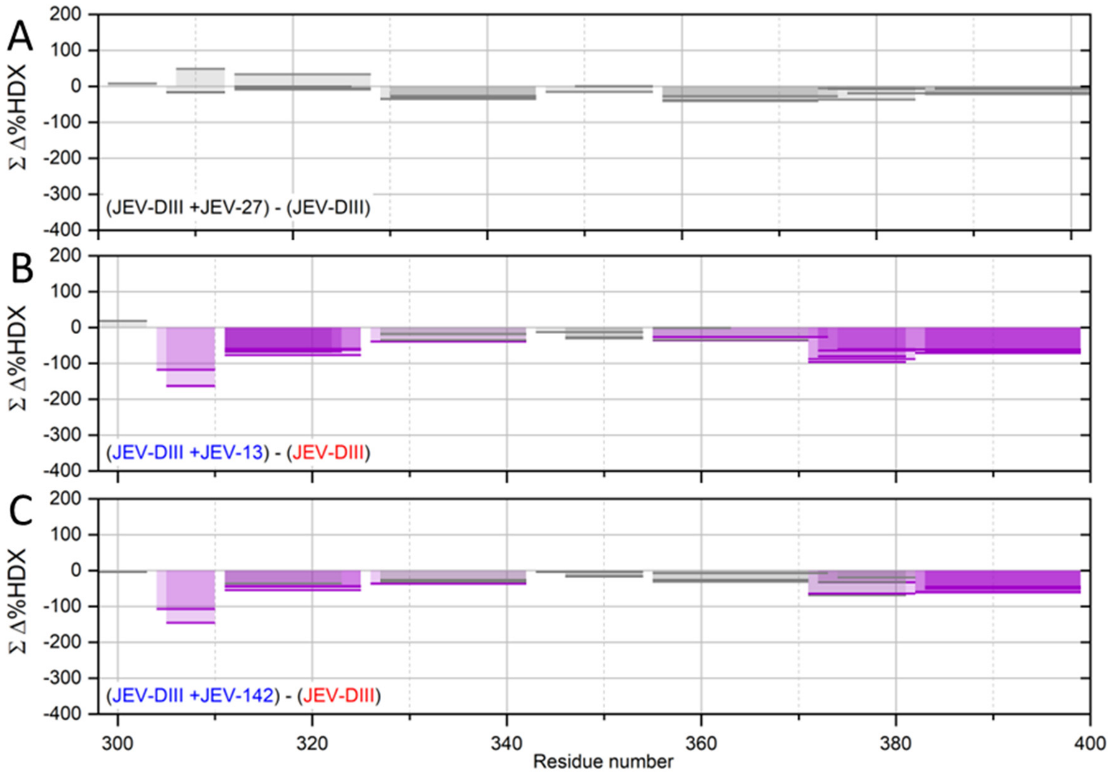

Figure S3: Differential Wood's plot for the JEV DIII in the presence and absence of antibodies JEV-27, JEV-13, and JEV-142. Cumulative sum of differences across all HDX time points for each peptide was calculated. Based on propagated error in HDX measurements, statistically protected peptides were highlighted. The gray bars depict peptides where there is no significant change in the bound vs. unbound states ($p = 0.01$) while violet bars indicate peptides exhibiting protection upon binding to antibody.
